# Supplementary material for: The Systems Biology Research Tool: evolvable open-source software
Source: BMC Syst Biol. 2008 Jun 29;2:55. doi: 10.1186/1752-0509-2-55 (PMC2446383; doi:10.1186/1752-0509-2-55)
Supplement: Additional file 1 — SBRT Archive. An archive of the current version of the Systems Biology Research Tool. [file 1752-0509-2-55-S1.zip › sbrt-1.4.0/doc/developers_guide/api/sbrt/kernel/math/algebra/SmdMatrix.html]

SmdMatrix


|  |  |  |
| --- | --- | --- |
| **Overview** \| **Package** \| **Class** \| **Use** \| **Tree** \| **Deprecated** \| **Index** \| **Help** | |  |
| **PREV CLASS**   **NEXT CLASS** | **FRAMES**    **NO FRAMES**     **All Classes** |  |
| SUMMARY: NESTED \| FIELD \| CONSTR \| METHOD | DETAIL: FIELD \| CONSTR \| METHOD |  |


---


## sbrt.kernel.math.algebra Interface SmdMatrix<R,C>

**Type Parameters:**: `R` - the row type.: `C` - the column type.

**All Superinterfaces:**: DoubleMatrix<R,C>, DoubleMatrixBuilder<R,C>, Matrix<R,C,java.lang.Double>, MatrixBuilder<R,C,java.lang.Double>, SparseDoubleMatrix<R,C>

**All Known Implementing Classes:**: AbstractSmdMatrix, HashMatrix, RowOrTreeMatrix, TreeMatrix

---

``` public interface SmdMatrix<R,C> extends DoubleMatrixBuilder<R,C>, SparseDoubleMatrix<R,C> ```

This interface is used to represent sparse mutable matrices
containing strictly double precision numbers as elements.

**Author:**
:   This interface was written and documented by
    Jeremiah Wright while in the Wagner lab.

---

| **Method Summary** | |
| --- | --- |
| `void` | `setElements(SparseDoubleMatrix<? extends R,? extends C> m)`             Sets the corresponding elements of this matrix to those of the provided matrix. |

**Methods inherited from interface sbrt.kernel.math.algebra.DoubleMatrixBuilder**

`setElement, setElements`

**Methods inherited from interface sbrt.kernel.math.algebra.MatrixBuilder**

`removeColumn, removeRow, setElement, setElements`

**Methods inherited from interface sbrt.kernel.math.algebra.Matrix**

`columns, equals, getColumnIndices, getRowIndices, hashCode, isColumnIndex, isEmpty, isRowIndex, rows, toString`

**Methods inherited from interface sbrt.kernel.math.algebra.DoubleMatrix**

`getElement, toLinearCombs`

**Methods inherited from interface sbrt.kernel.math.algebra.Matrix**

`columns, equals, getColumnIndices, getRowIndices, hashCode, isColumnIndex, isEmpty, isRowIndex, rows, toString`

**Methods inherited from interface sbrt.kernel.math.algebra.SparseDoubleMatrix**

`getColIndNzEl, getColumn, getRow, getRowIndNzEl`

**Methods inherited from interface sbrt.kernel.math.algebra.DoubleMatrix**

`getElement, toLinearCombs`

**Methods inherited from interface sbrt.kernel.math.algebra.Matrix**

`columns, equals, getColumnIndices, getRowIndices, hashCode, isColumnIndex, isEmpty, isRowIndex, rows, toString`

**Method Detail**

### setElements

```
void setElements(SparseDoubleMatrix<? extends R,? extends C> m)
```

:   Sets the corresponding elements of this matrix to
    those of the provided matrix.

    :   **Parameters:**: `m` - the matrix whose elements are to be copied into this matrix.


---


|  |  |  |
| --- | --- | --- |
| **Overview** \| **Package** \| **Class** \| **Use** \| **Tree** \| **Deprecated** \| **Index** \| **Help** | |  |
| **PREV CLASS**   **NEXT CLASS** | **FRAMES**    **NO FRAMES**     **All Classes** |  |
| SUMMARY: NESTED \| FIELD \| CONSTR \| METHOD | DETAIL: FIELD \| CONSTR \| METHOD |  |


---
